# Public Policy Versus Individual Rights in Childhood Obesity Interventions: Perspectives From the Arkansas Experience With Act 1220 of 2003

**Published:** 2011-08-15

**Authors:** Martha M. Phillips, Kevin Ryan, James M. Raczynski

**Affiliations:** University of Arkansas for Medical Sciences; University of Arkansas for Medical Sciences, Little Rock, Arkansas; University of Arkansas for Medical Sciences, Little Rock, Arkansas

## Abstract

Childhood obesity is a major public health problem. Experts recommend that prevention and control strategies include population-based policies. Arkansas Act 1220 of 2003 is one such initiative and provides examples of the tensions between individual rights and public policy. We discuss concerns raised during the implementation of Act 1220 related to the 2 primary areas in which they emerged: body mass index measurement and reporting to parents and issues related to vending machine access. We present data from the evaluation of Act 1220 that have been used to address concerns and other research findings and conclude with a short discussion of the tension between personal rights and public policy. States considering similar policy approaches should address these concerns during policy development, involve multiple stakeholder groups, establish the legal basis for public policies, and develop consensus on key elements.

## Introduction

Childhood obesity has rapidly become a major public health problem; rates may have leveled, but they have not declined (1). Medical costs for childhood obesity-related illness in the United States are estimated at more than $10 billion annually (2), and future medical costs for overweight adolescents may approach $46 billion (3). Left untreated, today's overweight adolescents are expected to experience 161 million years of life complicated by obesity, diabetes, and heart disease (3).

Given the enormity of the obesity burden, a collective response is needed for addressing obesity. Opportunities are available at multiple levels — communities, schools, industry, media, families, and individuals — to reduce the prevalence of obesity (4), and a complex-systems approach, encompassing multiple levels of a social-ecological model (5), is recommended by experts (6). Political discussions are dominated by consideration of the relative weight that should be given to personal responsibility and population approaches (1).

Population-based obesity control policies are recommended (7) to affect diverse population groups and to promote healthy physical activity and eating as default or normative behaviors (8). Policy proponents argue that society is obligated to protect children and other vulnerable populations from harm and ensure their right to safe and healthy environments (1).

Arkansas Act 1220 of 2003 (9) was among the first comprehensive legislative initiatives to combat childhood obesity. Its implementation provides an example of the controversies inherent in childhood obesity policy initiatives and the tensions between individual rights and public policy.

## Background of Arkansas Act 1220 of 2003

The development of Act 1220 has been described in detail elsewhere (10). Briefly, the impetus for the act can be traced to 2 conferences (10) attended by legislators in early 2002 that focused on reducing childhood obesity. Subsequently, the Arkansas House of Representatives speaker-elect requested that the Department of Health work with the Department of Education and other constituencies to draft a bill delineating school policy changes to reduce childhood obesity. This bill was introduced during the 2003 legislative session with strong support in both the state House and Senate and was passed into law quickly.

Act 1220 had 6 key elements: 1) annual measurement of body mass index (BMI) for public school children and a report of each child’s BMI and associated health risks sent to parents (this element was modified in 2007 to require BMI assessment only in kindergarten and even-numbered grades 2-10), 2) elimination of access to vending machines during school for elementary school students, 3) identification of funding to hire community health promotion specialists to work with schools and communities, 4) creation of a statewide Child Health Advisory Committee (CHAC) to recommend evidence-based school nutrition and physical activity regulations, 5) public reporting of vending contracts, and 6) establishment of school nutrition and physical activity advisory committees. CHAC deliberations led to regulations enacted by the state board of education (11), including restrictions on vending machine access in all public schools.

## Evaluation of the Implementation and Outcomes of Act 1220

Soon after Act 1220 became law, the Robert Wood Johnson Foundation (RWJF) funded the Arkansas Center for Health Improvement at the University of Arkansas for Medical Sciences (UAMS) to develop and analyze a statewide BMI database. RWJF also funded the UAMS Fay W. Boozman College of Public Health to conduct a process and impact evaluation of the act's implementation. The projects funded by these 2 distinct grants provide data relevant to the concerns surrounding individual rights versus public policy and how Arkansans viewed these concerns. The funding from RWJF for the evaluation will ultimately cover an 8.5-year evaluation period.

The evaluation used both quantitative and qualitative methods to obtain data from multiple sources, including principals, superintendents, randomly selected parents and adolescents (aged 14-18 y), minutes from CHAC and other relevant meetings, and perspectives from key stakeholders. Records review, key informant interviews, surveys, and telephone interviews all contributed data for the evaluation, described in detail elsewhere (10). Baseline data collection for the evaluation began in spring 2004, before implementation of any policy components, and will continue through 2012.

## Reaction to Act 1220

The act passed into law with little controversy, essentially unnoticed by people outside the legislature. Subsequent attention stimulated public concern (10) in 2 primary categories — concerns related to BMI measurement and reporting and those related to changes in vending machine access and contents — specifically to individual rights versus public policy concerns.

### Concerns related to BMI measurement and reporting

Act 1220 initially required public school children in grades kindergarten through 12 to have their BMI assessed annually, and reports to be sent to parents on report cards; however, this requirement was immediately modified by the legislature to require that confidential reports be sent to parents. Nonetheless, the following concerns continued to be reported:

1. **Informing parents of their children's weight status is unnecessary because they already know it**. Although parents commonly reported that they could recognize an overweight child, data indicate that even professionals have difficulty correctly classifying children's weight status (12). Evaluation data reveal that parents often do not correctly classify overweight children, although correct parental classification improved after Act 1220 implementation (13).
2. **BMI measurement and reporting violate confidentiality and invade privacy. Parents and school personnel both reported concerns in this area. **However, evaluation data gathered from parents, principals, and superintendents reveal a different attitude. Each year, a majority of parents reported being comfortable with receiving the BMI report (Table 1), and even in early years of implementation, school administrators rarely reported receiving calls or other contacts from parents about BMI measurements. By the third year of BMI measurement, a majority of principals (64%) and superintendents (54%) reported that they had no calls from parents about the measurements (14). Some vocal parents and school administrators, and even some members of the media, raised concerns about invasion of privacy from BMI measurement. However, with the Arkansas Center for Health Improvement's leadership, health, school, and professional communities collaborated early in the act's implementation to develop procedures for BMI assessment to ensure confidentiality (10). Evaluation data support the success of these efforts; in recent years, approximately three-fourths of parents reported comfort with confidentiality of the assessment and reporting processes, and students rarely reported embarrassment (Table 1). Parent and adolescent data demonstrate that most families do not have privacy concerns about BMI assessment/reporting.
3. **Schools have neither responsibility nor time for measuring BMI**. School administrators expressed concern that schools are primarily focused on education, not reducing obesity, and that taking time to measure BMI, in particular, reduces their time for education. Nonetheless, data reveal that school personnel acknowledge their responsibility for contributing to children's overall development (15), and very few principals and superintendents actually reported logistical or other problems with BMI assessment (14).
4. **Harm (eg, increased weight-based teasing, increased eating disorders, and negative emotional consequences) may occur because of BMI assessment**. Concerns emerged from both parents and health professionals about adverse consequences related to emotions, unhealthy diets, and increased eating disorders. However, these consequences have not materialized (10). Students reported a reduction in these anticipated negative consequences over time (Table 2), although confidence intervals were wide, largely because of limited sample sizes.

### Concerns related to vending machine access

The evaluation revealed the following concerns about individual versus public rights associated with vending machine changes:

1. **School budgets would be adversely affected**. School personnel expressed concern that their schools would lose revenue from reduced vending machine purchases. Although these revenues are unrestricted and therefore provide substantial flexibility in their use, 81% of schools reported vending revenues of less than $5,000 per year. Approximately 75% of schools reported stable or increased vending revenues between evaluation years 2004 and 2005 (15). Thus, vending revenues were low for most schools and apparently not affected by the legislation. Furthermore, other states have determined that vending machine revenues do not decline as healthy options increase (16).
2. **Students should not be forced to accept healthier options and will not purchase them when available.** Both parents and school personnel expressed concerns that students should not be forced to accept healthier options. However, evaluation data indicate that a majority of parents believe that schools should not have vending machines in middle and high schools (Table 3). Data also indicate that an increasing number of parents (59% in 2007, up from 51% in 2004) believe that schools should have only healthy options in vending machines, and most parents reported believing that schools should have at least a balance of healthy and unhealthy options. Although the evaluation did not assess student purchases, other research reports that students purchase healthy options when these are available (16).
3. **Students will get unhealthy options elsewhere if these options are unavailable at school.** Principals and superintendents, in particular, expressed the belief that having less access to unhealthy options in schools might cause students to purchase more unhealthy options outside of school (14). However, data from middle schools in Connecticut demonstrate that replacing low-nutrition items in schools with healthier options resulted in decreased student consumption in school of unhealthy beverages and salty snacks but no increase in unhealthy consumption at home (17). The study did not specifically assess out-of-school purchases but did demonstrate that low-nutrition food and beverage consumption does not necessarily increase, at least at home, with a shift toward healthier options in schools.

## Legal Rationales for and Against Government Actions in Addressing Obesity

The overarching legal concern raised with Act 1220 by proponents of individual rights was whether the legislature and, later, health and education departments, acted beyond their legal authority. Government entities have the sole, legitimate authority to pursue actions and initiatives intended to improve public health (1). Private entities pursue public health policies legitimately when acting as an agent of state or federal governments. However, the population perspective of public health programs and policies inevitably affects individual rights and freedoms (18). Maximizing public health protections may require restricting individuals' freedoms to behave in ways that are potentially deleterious to the population's health. For example, food safety regulations restrict the freedom of food growers, processors, transporters, and vendors to act without limits, but because of restrictions and requirements placed on these entities, the public enjoys safer foods. Having fewer restrictions maximizes individual freedom but may not acceptably guarantee the public's health. In contrast, more restrictions can reduce foodborne illnesses but lessen freedom of action by individuals and companies. From a societal perspective, although the health and safety of the public are valued, in the United States, an even higher value is typically placed on individual rights and freedoms.

### Government authority to act

Authority to protect the public's health rests primarily with state governments, although the federal government does have some responsibility in this area. State governments act under 2 primary types of legal authority: *parens patriae* power (state power to act for those who cannot care for themselves) and police power (state power to act in pursuit of the public's health, welfare, safety, and morals) (19). Although both types of authority allow the state to act to ensure public health, *parens patriae* authority typically has less impact than police power authority (20).

In addressing the individual and population burden of childhood obesity, Arkansas acted within its established authority to protect and promote the public's health (21). The effectiveness of this effort appears to have contributed to a decreasing prevalence of obesity in Arkansas without resulting in adverse consequences (22). These gains in public health came about, however, as a result of individual-level restrictions. Act 1220 limited student access to vending machines, mandated schools to disclose vending contracts, restricted their freedom to contract, and required students to complete BMI assessment without express parental consent. Although opposition has been modest, a limited but vocal group maintains that restricting individual freedoms, even given the public health goals, is an impermissible exercise of government authority.

### Individual rights and freedoms

In our federalist government system, individual rights and freedoms are guaranteed by the US Constitution, state constitutions, and federal and state laws. The US Constitution is the express descriptor of individual rights and limits the federal government's ability to act against them (23). Selected rights and clauses within rights are especially relevant to the context of individual rights. The rights of due process and equal protection, the Fourth Amendment protection against unreasonable search and seizure, and Article I's commerce and contracts clauses all place limits on the government's authority to act by expressly delineating individual rights and freedoms (19).

Individuals enjoy certain rights of privacy regarding their activities and personal information, established through federal and state laws (and promulgated through regulation and interpretative guidance). Two are particularly notable for this discussion: the Health Insurance Portability and Accountability Act of 1996 (HIPAA) and the Family Education Rights and Privacy Act of 1974 (FERPA) (19). Both HIPAA and FERPA safeguard the confidentiality of individual information against release to the government or other entities without explicit consent. However, these safeguards are not uniformly applied or always clearly understood, and their implementation can create conflict.

A consideration of HIPAA and FERPA clearly exemplifies the conflict between public health policy and individual rights. When aggregated, individual health data can guide policy makers and public officials in developing and evaluating responses to public health threats such as obesity. Yet, this aggregation is possible only through collection and release of individual-level information. In its original iteration, Act 1220 required schools to collect student BMIs and send reports to parents but did not address parental consent. The legislation was later amended to include a provision for parents to choose not to have their child assessed. However, the legislation does not address parental consent to include their child's information in data sets for further analysis.

### Extent of government authority to act against individual rights and freedoms

Thousands of federal and state laws and agency regulations direct the government to pursue public health initiatives. Numerous court decisions confirm use of government power in these instances, either through express grant at the federal level or under cover of police power and *parens patriae* at the state level. However, although no legal challenges to Act 1220 have emerged, the extent of this authority is not settled. The issues that policy makers and legal analysts debate include 1) the extent to which government authority to regulate interstate commerce through the Commerce Clause can support federal government action and 2) the boundaries of state police power (20). Thus, legal challenges to approaches such as Act 1220 may emerge as the debate about government authority continues.

## Conclusions and Implications for Public Health

Arkansas Act 1220 of 2003 was one of the first comprehensive legislative approaches to attempt to reduce childhood obesity through school-based policy changes. Its implementation raised substantial concerns related to public health policy versus individual rights. States considering similar legislation should address concerns that can emerge (eg, those related to BMI measurement and reporting and access to vending machines), involve multiple stakeholder groups, establish clearly the legal basis for public policies, and develop consensus regarding key policy elements. These efforts may help develop greater consensus about approaches to reducing childhood obesity and may lessen concerns.

## Figures and Tables

**Table 1:** Positive Parent and Student Responses to School-Based BMI Measurements and Reports, Arkansas, 2004-2008^a^

Response	Year, % Expressing Response				

	2004	2005	2006	2007	2008
**Parents**					
Comfortable with receiving a BMI report from child's school	70.3	67.1	67.6	60.8	63.4
Comfortable with confidentiality of BMI measurement and reporting processes	69.4	71.4	72.9	69.3	75.3
**Students^b^**					
Experience little or no embarrassment from BMI measurement process	89.8	91.1	92.3	86.3	88.7

Abbreviation: BMI, body mass index.

a Source: Evaluation of Arkansas Act 1220 (14).

b Adolescents aged 14-18 y.

**Table 2:** Negative Student^a^ Responses to School-Based BMI Measurements and Reports, Arkansas, 2004-2009^b^

Response	Year, % Expressing Response					

	2004	2005	2006	2007	2008	2009
Concern about weight	23.9	28.6	25.6	24.9	23.9	21.4
Teasing by peers because of weight	11.9	9.3	5.9	12.2	6.9	5.4
Beginning a diet within past 6 months	29.4	23.3	25.8	27.0	18.7	19.5
Taking diet pills	6.1	5.1	2.4	5.1	2.4	2.5

Abbreviation: BMI, body mass index.

a Adolescents aged 14-18 y.

b Source: Evaluation of Arkansas Act 1220 (14).

**Table 3:** Parent Opinions About Availability and Contents of Vending Machines in Arkansas Secondary Schools, 2004-2007^a^

Opinion	Year, % Expressing Opinion			

	2004	2005	2006	2007
Middle and high schools should not have vending machines at all.	57.0	58.1	60.6	51.8
Machines should have only healthy contents.	50.5	55.5	60.5	58.7
Machines should have both healthy and less healthy options.	42.9	39.4	35.1	38.5
No changes should be made to vending contents; they are fine as they are.	6.6	5.1	4.5	2.8

a Source: Evaluation of Arkansas Act 1220 (14).
